# Field estimation of deformation modulus of the soils by multichannel analysis of surface waves

**DOI:** 10.1016/j.dib.2019.103974

**Published:** 2019-05-08

**Authors:** V.V. Antipov, V.G. Ofrikhter

**Affiliations:** Perm National Research Polytechnic University (PNRPU), 29 Komsomolsky Prospekt, Perm, 614990, Russian Federation

**Keywords:** Wave analysis, Multichannel analysis of surface waves, MASW, Soil unit weight, Plate load test, PLT, Soil deformation modulus, Soil initial shear modulus, Velocity profile

## Abstract

The paper presents the processed results of Plate Load Tests and of Multichannel Analysis of Surface Waves for dispersive and semi-rocky soils at the sites with different soil conditions located in the Perm Region, Russian Federation. Unit weight and deformation modulus were calculated from the obtained data. The value of the data lies in their applicability for the prompt preliminary assessment of the site geotechnical situation.

**Table:** Specifications Table

Subject area	*Civil Engineering*
More specific subject area	*Environmental Geotechnics*
Type of data	*Tables, graphs, images (dispersive images and shear velocity profiles)*
How data was acquired	*Plate Load Tests (PLT), Multichannel Analysis of Surface Waves (MASW)*
Data format	*Filtered, analyzed*
Experimental factors	*PLT round plates* 600 cm^2^*, 2*500 cm^2^*and 5*000 cm^2^*. Active MASW with a 24-channel observation system with 0.5 m and 2 m receiver spacing. The sampling period and the total recording time were selected at the site by reconnaissance observations.*
Experimental features	*Testing soil types: sandfill, argillite-like clay, sand rock, clay, clayey sand, sandy clay, sand.*
Data source location	*Perm Region, Russian Federation, Sites No. 1-5 with different soil conditions*
Data accessibility	*The data is available within this article*
Related research article	*V.G. Ofrikhter, I.V. Ofrikhter, Investigation of municipal solid waste massif by method of multichannel analysis of surface waves, JGS Spec. Publ. 57 (2) (2015) 1956-1959.**http://doi.org/10.3208/jgssp.TC215-01**.* *V.G. Ofrikhter, I.V. Ofrikhter, M.A. Bezgodov, Results of field testing of municipal solid waste by combination of CPTU and MASW, Data in Brief 19 (2018) 883-889.**https://doi.org/10.1016/j.dib.2018.05.109**.*

**Table:** 

**Value of the data** • Methods outlined in the paper are a fast non-expensive approach that allows geotechnical engineers to carry out quick preliminary estimation of physical-mechanical properties of the soils and geotechnical situation on the surveyed sites; • MASW data can be used for rapid estimation of physical characteristics of the soils, in particular the soil unit weight; • MASW data can also be used to promptly assess the deformation modulus of the soils. Results were obtained by comparison of PLT and MASW data recorded at the same investigation points.

## 1 Data

The MASW results are presented in the summary Table 1 together with the soil unit weight calculations. Unit weights determined in the laboratory are presented for comparison. Calculated deformation moduli and initial shear moduli according to PLT and wave analysis are given in Table 2. Deformation modulus was calculated according to standard procedure recommended by GOST 20276-2012 [1] for the first four points of the load-settlement curve counting from initial pressure under plate.

**Table 1:** Summary table of the wave analysis results and data of unit weight calculation.

Site No.	Point No.	Soil type	*V*_s_, m/s	ρ, kg/m^3^	*G*_0_, MPa	*h*, m	*z*, m	GWT, m	*V*_s_, m	γ_calc_, kN/m^3^	γ_lab_, kN/m^3^
1	1	Sand fill	245	1826	109.64	1.5	1.5	–	245	19.59	17.9
2	1	Argillite-like clay	332	2010	221.57	3.5	11.5	1.5	332	19.27	19.7
	1	Sand rock	417	2040	354.88	>1.1	12.6	1.5	417	19.27	20.0
3	1	Clay	151	2112	48.16	0.5	0.5	3	151	18.61	20.7
4	1	Clayey sand	172	2040	60.38	>1.4	3	1.6	118	16.47	20.0
	2	Sandy clay	118	1918	26.71	0.7	3.1	1.9	547	21.99	18.8
5	1	Sand	142	1663	33.54	1	1	5	142	17.91	16.3

ρ is soil density; *V*_s_ is shear wave velocity; *G*_0_ is initial shear modulus of small strain; GWT is ground water table; *h* is soil layer thickness; *z* is layer base depth; *V*_s_ is shear wave velocity; γ_calc_ is soil unit weight; γ_lab_ is soil unit weight determined in the laboratory.

**Table 2:** Evaluation of deformation modulus by GOST 20276-2012 [1].

Site No.	Point No.	Soil type	GWT, m	*h*_pl_, m	*A*, cm^2^	*P*_n_, MPa	*P*_0_, MPa	*G*_0_, MPa	*E*, MPa	*m*	*E*_5000_, MPa
1	1	Sand fill	–	0	2500	0.25	0.1	109.64	24.24	1.06	25.70
2	1	Argillite-like clay	1.5	9.19	600	0.8	0.2	221.57	37.75	1.06	40.02
	1	Sand rock	1.5	11.7	600	0.8	0.2	354.88	58.22	1.06	61.72
3	1	Clay	3	0.1	600	0.2	0.05	48.16	5.92	1.2	7.10
4	1	Clayey sand	1.6	1.6	5000	0.125	0.05	60.38	9.52	1	9.52
	2	Sandy clay	1.9	2.4	5000	0.125	0.05	26.71	5.06	1	5.06
5	1	Sand	5	0.1	600	0.2	0.05	33.54	13.25	1.2	15.90

GWT is ground water table; *h*_pl_ is the plate level from the surface; *A* is the plate area; *P*_n_ is plate pressure corresponding to the fourth point of the linear part of the load-settlement curve; *P*_0_ is initial pressure corresponding to vertical intergranular stress from soil self-weight at the test level; *G*_0_ is initial shear modulus of small strains; *E* is PLT deformation; *m* is deformation modulus conversion factor; *E*_5000_ is calculated deformation modulus of 5000 cm^2^.

**Fig. 1:**
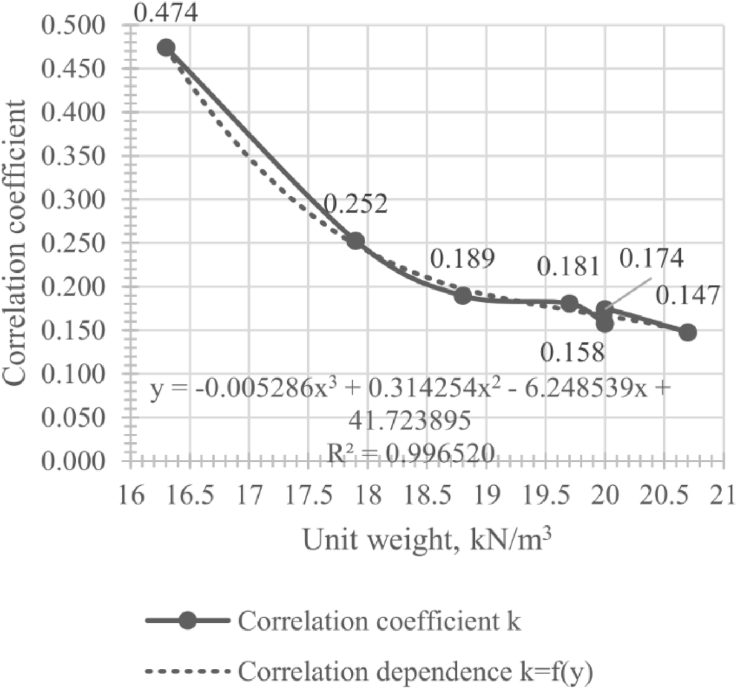
Unit weight - correlation coefficient.

Fig. 1 and Table 3 present correlation coefficients between deformation modulus and initial shear modulus. The correlation coefficient was calculated by the formula: *k* = *E*_5000_/*G*_0_; and next the dependency was obtained:

(1) $$k=-0.005286\gamma^{3}+0.314254\gamma^{2}-6.248539\gamma +41.723895;R^{2}=0.9965$$

where γ is soil unit weight, kN/m^3^; *k* is the correlation coefficient between MASW initial shear modulus and soil deformation modulus determined by formula (2):

(2) $$E=kG_{0}$$

**Table 3:** Unit weight - correlation coefficient data.

No.	Soil type	*G*_0_, MPa	*E*_5000_, MPa	γ_lab_, kN/m^3^	*k* = *E*_5000_/*G*_0_
1	Sand fill	33.54	15.90	16.3	0.474
2	Argillite-like clay	109.64	25.70	17.9	0.252
3	Sand rock	26.71	5.06	18.8	0.189
4	Clay	221.57	40.02	19.7	0.181
5	Clayey sand	60.38	9.52	20	0.158
6	Sandy clay	354.88	61.72	20	0.174
7	Sand	48.16	7.10	20.7	0.147

## 2 Experimental design, materials, and methods

### 2.1 Description of the sites

PLT tests and MASW surveys were performed at five sites with different soil conditions:

1. Site No. 1. Soil under the foundation slab:
  − Sand fill of fine homogeneous dense low moisture sand;
2. Site No. 2. Highway. Site beside a pillar of bridge crossing:
  − Medium strength loose fractured saturated argillite-like clay with pockets of low and medium strength sand rock;
  − Fine-grained loose fractured saturated sand rock of low and medium strength;
3. Site No. 3. Site of the former factory that is free of constructions:
  − Tough and medium-hard clay;
4. Site No. 4. Base of the foundation plate for a residential building:
  − Gray-brown areneceous fluid clayey sand with veins and pockets of 3–5 cm fine gray saturated sand and very soft brown clayey sand;
  − Dark-gray heavy silty very soft sandy clay with up to 15% inclusions of well-decomposed black organic matter;
5. Site No. 5. A test site of the chair “Construction operations and geotechnic” of PNRPU that is free of constructions:
  − Brown fine-grained sand.

Physical properties of the soils determined in the laboratory are presented in Table 4.

**Table 4:** Physical properties of the soils at testing sites.

Site No.	Point No.	Soil type	*w*	*w*_L_	*w*_P_	γ, kN/m^3^	γ_s_, kN/m^3^	γ_d_, kN/m^3^	*e*	*S*_r_
1	1	Sand fill	0.068	–	–	18.2	26.2	17.0	0.54	0.33
2	1	Argillite-like clay	0.170	0.34	0.14	20.4	26.2	17.4	0.50	0.89
	1	Sand rock	0.170	–	–	20.5	26.6	17.5	0.52	0.87
3	1	Clay	0.129	0.33	0.07	21.2	27.0	18.4	0.47	0.75
4	1	Clayey sand	0.296	0.24	0.18	20.2	27.0	15.6	0.73	1.09
	2	Sandy clay	0.299	0.35	0.19	18.8	26.4	14.5	0.82	0.96
5	1	Sand	0.099	–	–	16.3	25.1	14.7	0.69	0.36

*W* is water content; *W*_L_ is liquid limit; *W*_P_ is plastic limit; *I*_P_ is plasticity index; *I*_L_ is liquidity index; ρ is density; ρ_s_ is particles density; ρ_d_ is dry soil density; *e* is void ratio; *S*_r_ is degree of saturation.

Plate Load Tests were performed in accordance with the standard procedure set out in the State Standard [1]. The true value of the deformation modulus is taken as the modulus *E*_5000_ obtained for a plate of 5000 cm^2^ [2], [3]. Deformation modulus determined for the 600 cm^2^ plate was transformed to the module *E*_5000_ using formula (3) [4]:

(3) $$E_{5000}=E_{600}\cdot m$$

where *E*_600_ is deformation modulus for the 600 cm^2^ plate; *m* is conversion factor depending on the void ratio *e* according to Table 3 of [4].

According to Ref. [4], for the plates of other areas the coefficient *m* in Eq. (3) can be calculated by the expression from Annex B of [5]:

(4) $$m={\left(A_{5000}/A_{i}\right)}^{n/2}$$

where *A*_5000_ is the 5000 cm^2^ plate; *A*_i_ is the *i* cm^2^ plate area; *n* is reduction argument according to Annex B of [5], for silt-loam soil *n* = 0.15–0.3, for sandy soil *n* = 0.25–0.5.

Multichannel Analysis of Surface waves (MASW) is a non-expensive rapid non-invasive field method of wave analysis of the low velocity zone in the upper part of soil profile. The procedure of field survey and further data processing used by the authors is described in the papers [6], [7]. Optimum parameters were taken according to the papers [8], [9], [10], [11].

The obtained values of the S-wave velocities in the tested soil layers were used to calculate initial shear moduli from the expression [12]:

(5) $$G_{0}=\text{ρ}V_{s}^{2}$$

where ρ is soil density determined in laboratory tests, kg/m3; Vs is soil layer shear wave velocity, m/s.

It is worth noting that expression (6) proposed in paper [12] allows calculation of the soil unit weight with values of S-wave velocities and depth:

(6) $$\gamma =8.32\mathrm{lg}\left(V_{s}\right)-1.61\mathrm{lg}\left(z\right)$$

where γ is unit weight of the soil layer, kN/m^3^; *z* is layer base depth, m.
